# HIV-1C *env* and *gag* Variation in the Cerebrospinal Fluid and Plasma of Patients with HIV-Associated Cryptococcal Meningitis in Botswana

**DOI:** 10.3390/v12121404

**Published:** 2020-12-07

**Authors:** Nametso Kelentse, Sikhulile Moyo, Mompati L. Mogwele, Doreen Ditshwanelo, Baitshepi Mokaleng, Natasha O. Moraka, Kwana Lechiile, Tshepo B. Leeme, David S. Lawrence, Rosemary Musonda, Ishmael Kasvosve, Thomas S. Harrison, Joseph N. Jarvis, Simani Gaseitsiwe

**Affiliations:** 1 Botswana Harvard AIDS Institute Partnership, Gaborone, Botswana; thobanenametso@gmail.com (N.K.); smoyo@bhp.org.bw (S.M.); mmogwele@bhp.org.bw (M.L.M.); dditshwanelo@bhp.org.bw (D.D.); bmokaleng@bhp.org.bw (B.M.); nmoraka@bhp.org.bw (N.O.M.); klechiile@bhp.org.bw (K.L.); tleeme@bhp.org.bw (T.B.L.); david.s.lawrence@lshtm.ac.uk (D.S.L.); rmusonda@bhp.org.bw (R.M.); joseph.jarvis@lshtm.ac.uk (J.N.J.); 2 School of Allied Health Professions, Faculty of Health Sciences, University of Botswana, Gaborone, Botswana; kasvosvei@ub.ac.bw; 3 Department of Immunology and Infectious Diseases, Harvard T.H. Chan School of Public Health, Boston, MA 02115, USA; 4 Department of Biological Sciences, University of Botswana, Gaborone, Botswana; 5 Department of Pathology, Stellenbosch University, Stellenbosch 7505, South Africa; 6 Botswana-University of Pennsylvania Partnership, Gaborone, Botswana; 7 Department of Clinical Research, Faculty of Infectious and Tropical Diseases, The London School of Hygiene and Tropical Medicine, London WC1E 7HT, UK; 8 Centre for Global Health, Institute for Infection and Immunity, St. George’s University of London, London SW17 0RE, UK; tharriso@sgul.ac.uk; 9 Department of Medicine, Division of Infectious Diseases, Perelman School of Medicine, University of Pennsylvania, Philadelphia, PA 19104, USA

**Keywords:** Botswana, CCR5, CXCR4, cerebrospinal fluid, co-receptor, cryptococcal meningitis, escape mutations, human immunodeficiency virus (HIV), plasma

## Abstract

HIV-1 compartmentalization in reservoir sites remains a barrier to complete HIV eradication. It is unclear whether there is variation in HIV-1 *env* and *gag* between cerebrospinal fluid (CSF) and plasma of individuals with HIV-associated cryptococcal meningitis (CM). We compared HIV-1 *env* characteristics and the *gag* cytotoxic T-lymphocyte (CTL) escape mutations from CSF and plasma samples. Employing population-based Sanger sequencing, we sequenced HIV-1 *env* from CSF of 25 patients and plasma of 26 patients. For *gag*, 15 CSF and 21 plasma samples were successfully sequenced. Of these, 18 and 9 were paired *env* and *gag* CSF/plasma samples, respectively. There was no statistically significant difference in the proportion of CCR5-using strains in the CSF and plasma, (*p* = 0.50). Discordant CSF/plasma virus co-receptor use was found in 2/18 pairs (11.1%). The polymorphisms in the HIV-1 V3 loop were concordant between the two compartments. From the HIV-1 *gag* sequences, three pairs had discordant CTL escape mutations in three different epitopes of the nine analyzed. These findings suggest little variation in the HIV-1 *env* between plasma and CSF and that the CCR5-using strains predominate in both compartments. HIV-1 *gag* CTL escape mutations also displayed little variation in CSF and plasma suggesting similar CTL selective pressure.

## 1. Introduction

Early in the course of infection, the human immunodeficiency virus (HIV) can cross the blood-brain-barrier and infect the central nervous system (CNS) [1,2]. Once in the CNS, restriction of viral gene flow can occur causing variability in the HIV viral strains found in the CNS and peripheral blood, known as compartmentalization [3]. Although the initiation of antiretroviral therapy (ART) early after HIV infection can reduce viral diversity significantly, compartmentalized quasispecies are still observed in the CNS [4]. It has been postulated that distinct viral variants arise as a result of differential immune pressure and/or drug pressure between the compartments. Variation in viral diversity in the CNS and peripheral blood has potentially important implications for HIV drug treatment and vaccine development and is considered to be a key barrier to successful HIV eradication [5]. Opportunistic infections of the CNS such as tuberculosis meningitis (TBM) have been shown to play a critical role in HIV compartmentalization [6]; however, there is limited evidence on whether or not cryptococcal meningitis (CM) have similar effects. Previous studies have shown that inflammation of the meninges caused by CM may result in the disruption of the blood-brain barrier (BBB), thereby causing the HIV-infected cells to easily penetrate the barrier into the CNS [7,8]. Once in the CNS, the virus may be subjected to different immune and/or drug pressure; however, whether or not the CNS quasispecies is distinct from those from peripheral blood is still not confirmed.

Several studies have described compartmentalization in the cerebrospinal fluid (CSF)-derived HIV-1 *env* [9,10]. The HIV-1 *env* contains two glycoproteins, the gp41 and gp120. The gp120 is a surface glycoprotein that is used by the virus to attach to the host CD4 receptors and co-receptors CCR5 or CXCR4 for cell entry [11,12]. The selection of the type of co-receptor used for entry depends on the stage of the HIV infection. CCR5-using strains are found during the early stages of HIV infection while CXCR4-using strains tend to occur during chronic stages of HIV infection [13,14]. Prior studies looking at HIV-1 strains in the CNS have found that the majority are CCR5-using strains [1,15]. However, patients with HIV-associated CM may be expected to harbour some CXCR4-using strains due to the association of CM with advanced HIV disease [16]. Determination of viral tropism may be of clinical relevance as it determines whether patients are susceptible to CCR5-antagonist drugs such as Maraviroc that are used in salvage therapy in patients who are failing multiple antiretroviral drugs (ARVs) [17]. HIV co-receptor use in the CNS can differ from plasma; however, mechanisms that drive this intra-patient HIV genetic variation are still unclear. Some studies have reported selection pressure imposed by the immune system and preferential tropism as the main drivers [10]. Quasispecies compartmentalization of HIV-1 *env* can also affect viral infectivity and response to broadly neutralizing antibodies [18]. This usually occurs when there is a difference in the number of potential N-linked glycosylation sites (PNGSs) between two compartments [19]. PNGSs are used by the virus to escape immune evasion through the addition of glycans (N-linked polysaccharide chains) to the HIV-1 *env* glycoprotein creating a glycan shield that restricts the binding of neutralizing antibodies to their epitopes [18].

Distinct HIV-1 *gag* viral variants in the CSF and plasma have also been reported in previous studies and this usually occurs due to positive selection from host cytotoxic T-lymphocytes (CTL) response [20,21]. This concept has been explored in prior studies using the simian immunodeficiency virus (SIV) infection of macaque models. Variation in the mutations occurring in the two compartments indicates that compartmentalization in the brain may lead to the persistence of immune escape mutants in a CNS reservoir, with potential implications for therapeutic vaccines and HIV cure strategies [22]. In contrast, findings from a study investigating the early emergence of SIV *gag* K165R escape mutations within the Mane-A*10-restricted KP9 *gag* epitope in plasma and CSF of ARV treated SIV-infected macaques showed the development of K165R SIV *gag* escape mutation in both CSF and plasma compartments [21]. 

These observations prompted us to examine whether *gag* and *env* regions of the HIV-1 genome are genetically distinct in CSF and plasma of individuals with HIV-associated CM. We compared viral tropism, the number of PNGSs and amino acid polymorphisms in the V2, V3, and V4 loops of HIV-1 gp120 between CSF and plasma, and evaluated HIV-1 *gag*-associated CTL escape mutations in the CSF and plasma.

## 2. Materials and Methods

### 2.1. Study Population

We conducted a cross-sectional study using patient data and archived samples from participants who were part of the “Ambisome Therapy Induction Optimization for Cryptococcal Meningitis” (AMBITION-cm) phase II clinical trial that was carried out between October 2014 and September 2016 with 80 participants from Princess Marina Hospital (Gaborone, Botswana) and Bugando Medical Centre and Seko Toure Hospital (Mwanza, Tanzania) [23]. All AMBITION-cm participants were confirmed to have HIV-1 by a rapid diagnostic test before enrolment or evidence of a previously documented positive test or detectable HIV-1 viral load (VL). They also tested positive for CM infection using India Ink or cryptococcal antigen in the CSF. Our study utilized CSF and plasma samples from 41 out of the 60 participants recruited in the Gaborone site and these selected samples were collected either on day 3 or 7 of the study trial dependent on availability.

### 2.2. Ethical Consideration

The study was approved by the University of Botswana Institutional Review Board (IRB) and a research permit was obtained from the Botswana Ministry of Health and Wellness (HPDME: 13/18/1). All participants had been previously consented to the use of their stored samples for research related to the pathophysiology of HIV-associated CM in the parent study (ISRCTN#10248064).

### 2.3. HIV-1 RNA Quantification and Extraction

HIV-1 VL was measured in plasma and CSF using Abbott m2000rt/m2000sp assay (Abbott Laboratories, Abbott Park, IL, USA) with a lower limit of detection of 40 copies/mL. HIV-1 RNA was extracted from 140 μL of plasma and CSF cell-free supernatant using a QIAamp RNA extraction mini kit (Qiagen, Hilden, Germany) according to the manufacturer’s instructions.

### 2.4. HIV-1 DNA Amplification and Sequencing

The gp120 region of the HIV-1 *env* was amplified in a one-step polymerase chain reaction (PCR) using a SuperScript™ III One-Step RT-PCR System with Platinum™ Taq DNA Polymerase (Thermofisher Scientific, Waltham, MA, USA) according to the manufacturer’s instruction. The first-round primers used were (AC-env; 5′-CAGATGCATGAGGATATAATCA-3′; HXB2 position 6531–6552) and (ED12m; 5′-AGTGCTTCCTTGCTGCTCCCAA-3′; HXB2 position 7811–7791). A KAPA HiFi Hot-Start Ready Mix kit (KAPA Biosystems, Woburn, MA, USA) was used for second-round PCR according to the manufacturer’s instructions. Equal volumes of 10 μM forward primer (ED5; 5′-ATGGGATCAAAGCCTAAAGCCATGTG-3′; HXB2 position 6556–6581) and 10 μM reverse primer (ED12; 5′-AGTGCTTCCTGCTGCTCCCAAGAACCCAAG-3′; HXB2 positions 7851–7822) were used in this reaction.

Complementary DNA (cDNA) of HIV-1 *gag* was synthesized using a Transcriptor one-step PCR kit (Roche Applied Science, Penzberg, Germany). Briefly, 10 μL of the RNA template was added to 15 μL of a master mix containing 5 μL of 5 × Transcriptor one-step buffer, 2.5 μL primer mix, 0.5 μL Transcriptor enzyme mix and 7 μL of nuclease-free water. A primer mix used in the reaction contained 10 μM forward primer (F2NST; 5′-GCG GAG GCT AGA AGG AGA GAG ATG G-3′; HXB2 positions 769–793) and 10 μM reverse primer (1448L; 5′-AGG GGT CGC TGC CAA AGA GTG ATT-3′; HXB2 positions 2258–2291). The PCR cycling conditions were performed as follows; Reverse transcription at 50 °C for 30 min, Initial denaturation at 94 °C for 7 min, then 10 cycles of the second denaturation at 94 °C for 10 s, annealing at 55 °C for 30 s and elongation at 68 °C for 2 min. This was followed by 35 cycles of denaturation at 94 °C for 10 s, the annealing temperature of 55.5 °C for 30 s, elongation at 68 °C for 2 min increasing by 10 s each cycle, then final elongation for 5 min at 68 °C followed by cooling at 4 °C. The KAPA HiFi Hot-Start Ready Mix kit (KAPA Biosystems, Woburn, MA, USA) was used in a second-round PCR as described by the manufacturer’s instructions. A primer mix consisting of 10 μM forward primer (GAG-5U; 5′-GTG CGA GAG CGT CAA TAT TAA GAG-3′; HXB2 positions 794–814) and 10 μM reverse primer (1445L; 5′-GGT CGC TGC CAA AGA GTG ATT-3′; HXB2 positions 2258–2278) was used in the reaction.

The *gag* and *env* second-round amplicons were loaded on a 1% agarose gel in Tris-Borate-EDTA (TBE) buffer and ran at 100 V for 45 min to confirm amplification success and verify amplicons quality. The amplicons were purified and sequenced using the big dye chemistry (Big Dye Terminator sequencing kit) according to the manufacturer’s protocol (Applied Biosystems, Foster City, CA, USA). The sequences generated were then purified using ZR DNA sequencing clean-up kit™ (Zymo Research, Pretoria, South Africa) then resolved on the ABI Prism 3130xl genetic analyzer (Applied Biosystems, Foster City, CA, USA).

### 2.5. Phylogenetic Analyses

The HIV-1 *env* and *gag* sequences were manually edited using Sequencher software version 5.0 (Gene codes Corporation, Ann Arbor, MI, USA). The HIV-1 *gag* sequences were aligned with the HXB2 reference strain in Bioedit version 7 software. The variable loop 2, 3 and 4 (V2, V3 and V4) of HIV-1 *env* were also aligned with HXB2 and consensus sequence for subtype C. We then constructed a maximum-likelihood phylogenetic tree for HIV-1 *gag* and *env* using PhyML version 3.0 with 1000 bootstrap replicates to check for contamination and relatedness [24]. Sequences were subtyped by online tools REGA HIV-1 subtyping tool ver. 3 and Context-based Modeling for Expeditious Typing (COMET HIV-1) [25,26]. The sequences were submitted to GenBank, (accession numbers: MW280355-MW280440).

### 2.6. Prediction of Co-Receptor Usage

The HIV-1 *env* V3 loop sequences were analyzed for co-receptor usage using three algorithms: Geno2Pheno (https://coreceptor.geno2pheno.org) with a false positive rate of 5%, WebPSSM subtype C sinsi (https://indra.mullins.microbiol.washington.edu/webpssm/) and a combination of 11/25 and net charge rule. For 11/25 and net charge combination rule, CXCR4 usage was determined using the following criteria: (a) a sequence having amino acid R/K at position 11 and/or K at position 25; (b) amino acid R at position 25 and a net charge of ≥ +5; or (c) net charge of ≥ +6. The amino acid net charge was calculated by adding all positive and negative charges within the V3 loop. Lysine and Arginine (K and R) are positively charged hence scored as +1 whilst the negatively charged Aspartate and Glutamate (D and E) were scored as −1. For sequences with mixed bases, all possible permutations were assessed and the combination with the highest charge was used for predicting tropism [27]. If two or three methods predict the same tropism, this was concluded to be the tropism (final tropism) for that particular strain.

### 2.7. Identification of N-Linked Glycosylation Sites

The V2, V3, and V4 loop sequences of HIV-1 gp120 were used to identify PNGSs using the N-Glycosite tool in the Los Alamos HIV sequence database (https://www.hiv.lanl.gov/content/sequence/GLYCOSITE/glycosite.html). This tool identified the PNGSs in the variable loop whose patterns were N-X-[S or T], where X is any amino acid except proline.

### 2.8. Identification of HIV-1 gag Associated Mutations

HIV-1 *gag* sequences from plasma and CSF were analyzed for HIV-1 CTL escape mutations that are documented in the Los Alamos HIV Immunology Database (https://www.hiv.lanl.gov/content/immunology/variants/ctl_variant.html). In this study, we could not do any human leukocyte antigen (HLA) typing; however, relevant epitopes with HLA restrictions were selected from the epitope database of Los Alamos HIV Immunology Database and used to identify variations within or flanking known CTL epitopes as well as compensatory mutations in the HIV-1 *gag* region. The epitopes analyzed were TL9, GL9, RM9, KK10, NY10, ISW9, KF11, TW10, and QW9. These epitopes are restricted by HLAs that have a combined prevalence of 26.2% in Botswana [28].

### 2.9. Statistical Analysis

A McNemar test was used to calculate the difference in the proportion of CCR5-using strains in CSF and plasma. The difference in amino acid sequence length and PNGS between CSF and plasma was calculated using a Wilcoxon signed-rank test. The *p*-values < 0.05 were considered to be statistically significant. All analysis was performed using R version 3.6.0 [29]. 

## 3. Results

### 3.1. Patient Characteristics

Out of the 41 participants in our study, 59.5% were male and the participants’ median age was 38 (interquartile range (IQR): 31–44) years. The majority (78.5%) of the individuals were ART-naïve with a median CD4+ T-cell count of 28 (IQR: 10–44) cells/uL. The participants had median plasma and CSF HIV-1 VL of 5.1 (IQR: 4.8–5.7) and 4.6 (IQR: 3.7–4.9) copies/mL respectively, median CSF white blood cell count of 10 (3.0–65.8) cells/μL and median fungal burden of 5.0 (IQR: 4.1–5.7) log_10_ CFU/mL (Table 1).

### 3.2. Phylogenetic Analysis

**Figure 1:**
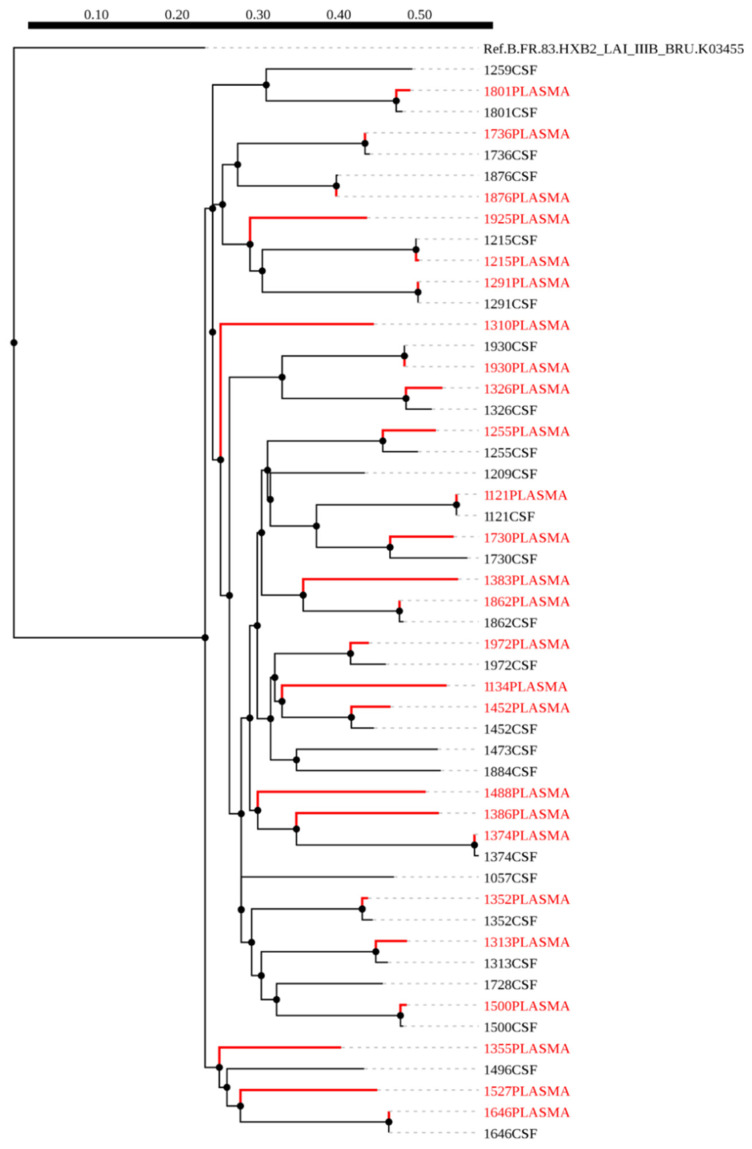
A maximum likelihood phylogenetic tree of HIV-1 *env* sequences from CSF and plasma samples of individuals with HIV-associated cryptococcal meningitis (CM). The distance scale is shown by the numbers at the top of the tree that represents the number of nucleotide substitutions per site. The branch length indicates genetic change.

**Figure 2:**
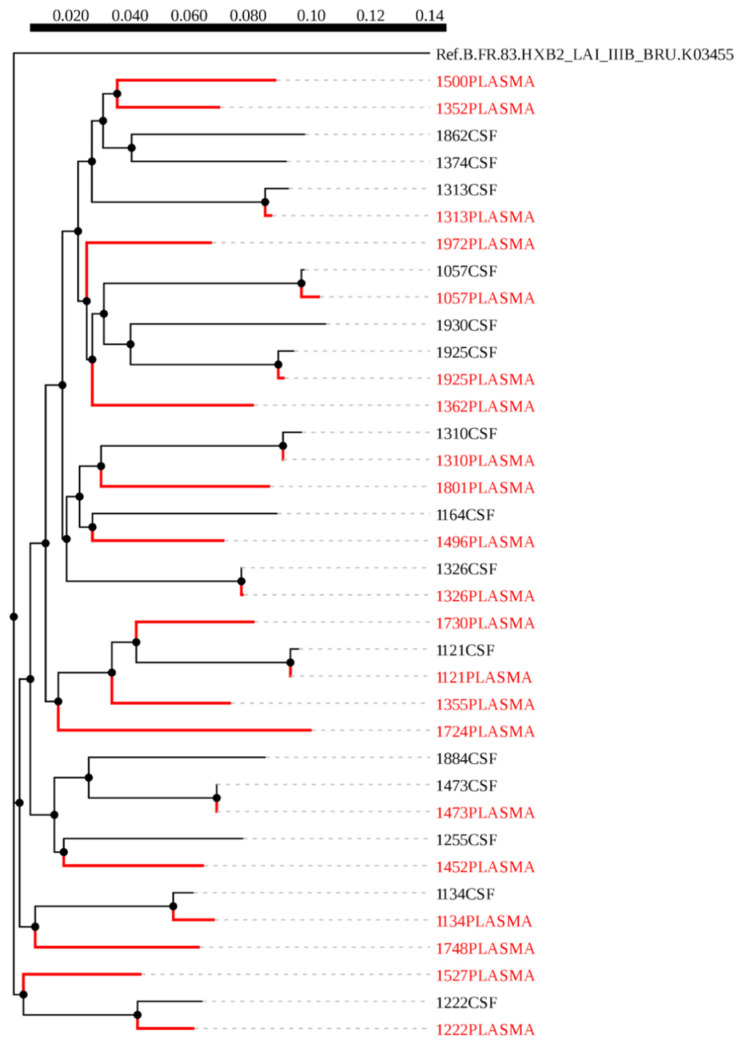
A maximum likelihood phylogenetic tree of HIV-1 gag sequences from cerebrospinal fluid (CSF) and plasma samples of individuals with HIV-associated CM. The distance scale is shown by the numbers at the top of the tree that represents the number of nucleotide substitutions per site. The branch length indicates genetic change.

A maximum-likelihood phylogenetic tree was constructed using 51 HIV-1 *env* sequences from plasma (red) and CSF (black), of which 18 were paired sequences and the rest were unpaired (Figure 1). We did not observe any form of contamination in our sequences as all the sequences from plasma and CSF of the same individuals paired accordingly with higher bootstrap values. We also constructed a phylogenetic tree from 36 HIV-1 *gag* sequences, of these, nine were CSF (black) and plasma (red) pairs (Figure 2). The CSF and plasma sequences from the same individual clustered with a bootstrap value of 100% confirming sequence purity. The phylogenetic trees from both HIV-1 *env* and *gag* sequences revealed that all the sequences generated were subtype C. 

### 3.3. HIV-1 Co-Receptor Usage in CSF and Plasma

We assessed the co-receptor usage in plasma and CSF using three different algorithms namely Geno2pheno, PSSM and 11/25 rule. We then concluded on the final tropism based on the three methods. From the 41 participants, we successfully sequenced 26 and 25 HIV-1 *env* sequences from plasma and CSF respectively, and these sequences were analyzed for co-receptor usage. Of these, 21 (80.8%) plasma and 21 (84%) CSF sequences had CCR5-using strains and the remaining were CXCR4-using strains.

**Figure 3:**
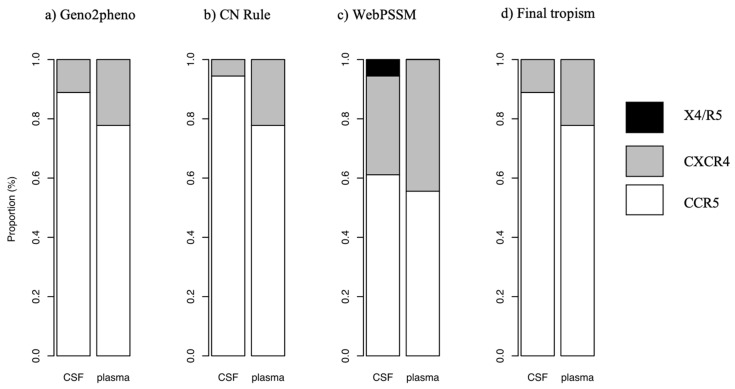
Comparison of co-receptor usage in CSF and plasma using (**a**) Geno2Pheno, (**b**) 11/25 rule (CN Rule) and (**c**) PSSM as well as the (**d**) final tropism based on the three methods.

The sequences obtained formed 18 pairs from plasma and CSF and the proportion of CCR5 using strains obtained using the three algorithms are reported in Figure 3. The proportion of CCR5-using strains obtained using Geno2pheno was 0.89 and 0.78 in the CSF and plasma, respectively, with the remaining being CXCR4-using strains. According to the 11/25 rule, the proportion of CCR5-using strains was 0.94 and 0.78 and CXCR4-using strains were 0.06 and 0.22 in CSF and plasma, respectively. Using the PSSM method, the proportion of CCR5-using strains, CXCR4-using strains and dual tropism (CXCR4/CCR5-using strains) was 0.61, 0.33 and 0.06 in the CSF, respectively and 0.66, 0.44 in plasma, respectively. The proportion of CCR5-using strains (final tropism) was 0.78 (95% CI: 0.52–0.94) in plasma and 0.89 (95% CI: 0.65–0.99) in the CSF and the difference was not significant between the two compartments (*p* = 0.5). CSF/plasma viral tropism discordance was found in 11.1% (95% CI: 0.1–3.4). The individuals who showed viral tropism discordance were both ART-naïve.

### 3.4. PNGSs in HIV-1 V2, V3 and V4 Loop Derived from CSF and Plasma Samples

**Figure 4:**
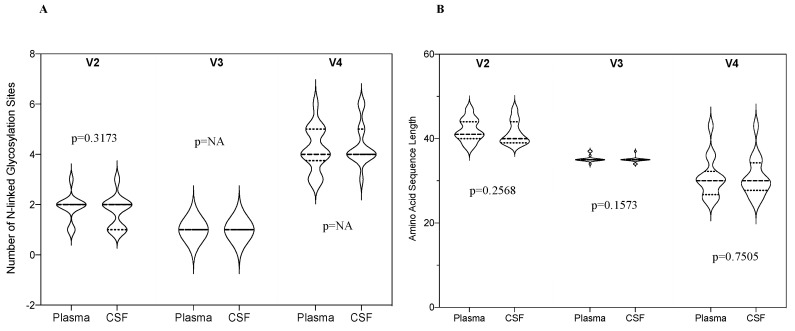
(**A**) A violin plot showing the median number of N-linked glycosylation sites in HIV-1 *env* V2, V3 and V4 loops in the CSF and plasma; (**B**) A violin plot showing amino acid sequence length in HIV-1 *env* V2, V3 and V4 loops in the CSF and plasma.

We compared the proportion of PNGSs in the three variable loops (V2, V3 and V4) in paired CSF and plasma samples. There was no significant difference in the number of V2 PNGSs between CSF and plasma (*p* = 0.32), Figure 4. There was no variation in the V3 and V4 hence *p*-value could not be calculated. We also compared the amino acid sequence length in the three variable loops and there was also no significant difference between the CSF and plasma (*p* = 0.26, 0.16 and 0.75 for V2, V3 and V4, respectively).

**Figure 5:**
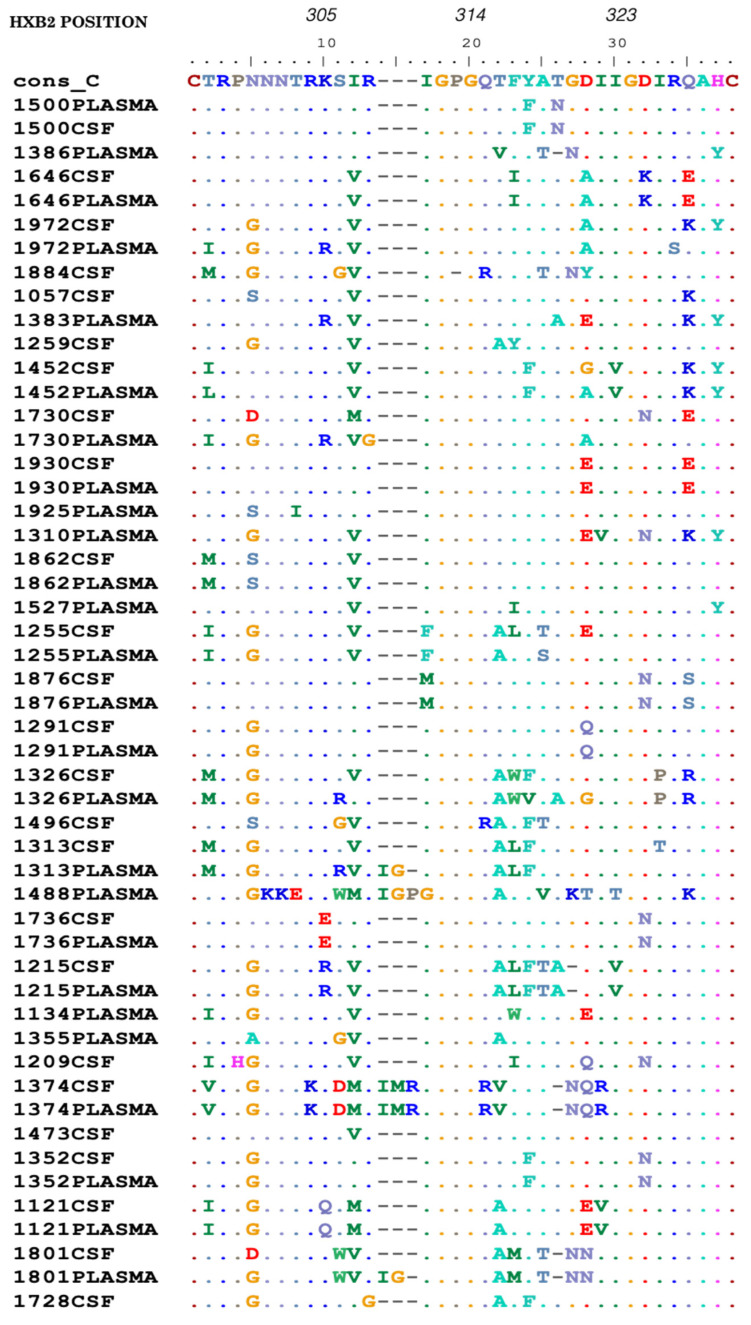
An alignment showing the HIV-1 gp120 V3 amino acids in CSF and plasma. Sequences are numbered relative to HXB2 and the dots represent amino acids identical to the consensus C sequence while dashes represent gaps.

The PNGS at position 301 in the HIV-1 *env* V3 loop was conserved in all paired CSF and plasma samples shown in Figure 5. All glycans at position 160 of the HIV-1 *env* V2 region (N160) were conserved in all paired samples except for one sequence (ID: 1452) from CSF that had lost its glycan site. The PNGS for the V2 loop are provided in Figure S1. In the V4 loop, we analyzed the PNGSs at positions 386 and 392 and we observed a loss of glycan site at position 392 in CSF of sequence 1255 and 1352 plasma. At position 386, Aspartate (D) was observed in sequence 1374 plasma and CSF that lead to the loss of glycan site. The PNGSs for the V4 loop are shown in Figure S2.

### 3.5. Mutations in the V3 Loop Region of HIV-1 gp120 in CSF and Plasma

We compared polymorphisms in the significant positions of the HIV-1 V3 loop from CSF and plasma. We had an overall of 51 sequences with 26 from plasma and 25 from CSF. We evaluated the prevalence of mutations A316T (position 22 in Figure 5) and I323V (position 30 in Figure 5) that has been previously associated with Maraviroc resistance in subtype B [30]. A316T was found in 61.5% (16/26) of plasma and 60.0% (15/25) CSF sequences (Figure 5). The prevalence of I323V in plasma and CSF was 7.7% (2/26) and 8.0% (2/25), respectively. For paired sequences, we observed A316T and I323V mutations in 61.1% (11/18) and 11.1% (2/18) pairs, respectively, and these mutations were concordant between the two compartments. 

### 3.6. HIV-1 gag-Associated Mutations in CSF and Plasma

A total of 36 sequences from HIV-1 *gag* were obtained from the samples, 21 were from plasma and 15 were obtained from CSF. We analyzed HIV-1 CTL escape and compensatory mutations in these sequences using nine selected epitopes with HLA restrictions and these are reported in Table 2. The most frequent mutation was I147L observed in 47.2% (17/36) sequences. T242N mutation that is known for reducing replication capacity was observed in only 5.6% (2/36) sequences. Out of the 36 sequences, we derived nine matched pairs of CSF and plasma and compared the mutations between the pairs. The CTL escape mutation discordance occurred in 3 different CSF/plasma pairs, in three out of the nine different epitopes analyzed.

## 4. Discussion

HIV compartmentalization in the CNS has been intensively described in ART experienced individuals due to a report of poor penetration of some ARVs through the BBB into the CNS leading to the emergence of discordant drug resistance mutations in the CNS and peripheral blood [31,32,33,34]. In this study, we discussed HIV viral compartmentalization in ART naïve individuals as it may highlight impact of host immune pressure in HIV compartmentalization. We, therefore, analysed partial *env* and *gag* genes of the HIV-1 genome in participants with HIV-associated CM. We had initially hypothesized that many of these isolates would be CXCR4-using strains since the participants are at an advanced stage of HIV infection as shown by the presence of CM and low CD4+ T-cell count; however, a high number of CCR5-using strains were observed in both compartments with no statistically significant difference in the proportion of CCR5-using strains in plasma and CSF (*p* = 0.5). This is consistent with other studies showing that the majority of individuals with CM or advanced HIV disease harboured CCR5-using strains in the CSF and plasma despite the chronicity of the HIV infection or presence of CM [9,10] and with data showing that unlike in HIV-1B, CCR5-using strains predominate in HIV-1C even though the individuals are at an advanced stage of the HIV infection [35].

The prevalence of CSF/plasma viral tropism discordance in our study was 11.1%. These individuals were ART-naïve; however, due to the small sample size, we could not establish implications of ART status on HIV discordance between the CSF and plasma compartment. Previous studies have also shown relatively little discordance in the two compartments and a slightly higher prevalence of CCR5-using strains in the CSF than in plasma [9,36,37]. The predominance of CCR5-using strains in these studies was not based on the chronicity of the HIV infection but rather functional compartmentalization of the variants between plasma and CSF. Few CXCR4-using strains were observed in the CSF, and the CSF was predominantly populated by CCR5-using strains suggesting an inability of the CXCR4-using strains to replicate effectively in the CNS macrophages and microglial cells, and a possibility of limited movement of infected cells from the peripheral blood into the CNS [36,38].

Prior studies have shown a difference in the average number of glycosylation sites between plasma and CSF as a result of potential selection pressure from neutralizing antibodies [39]. However, in our study, we did not find any statistically significant difference between the two compartments, which may be due to the relatively small sample size of our study. We analyzed position 386 of the *env* gp120 V4 loop that is associated with increased sensitivity to neutralizing antibodies if the glycan at this position is removed [40]. This loss increases the exposure of the b12 epitope overlapping the CD4 binding sites on the HIV-1 gp120 region consequently increasing sensitivity to neutralizing antibodies [40]. The D386 variant has also been associated with HIV-associated dementia (HAD) and an enhanced macrophage tropism that allows the virus to enter cells that are even expressing low levels of CD4 and CCR5 and this, in turn, increases replication preferentially in the CNS [40]. Due to this concept, we expected to see more of D386 in the CSF than plasma; however, in our sequences, a glycan at position 386 in the HIV-1 *env* V4 region was removed in CSF and plasma sequence in only one participant. There are conflicting data on whether the sequon for epitope 2G12 in the V4 region including the N392 is highly conserved or not due to one study showing high conservation while another study reported this site to be poorly conserved [41,42]. In our study, this sequon was only removed in CSF sequence 1255 and 1352 plasma, which confirms that this region is conserved and can appear distinct in two different compartments.

All PNGSs at position 301 in the HIV-1 *env* V3 loop were conserved and were concordant in paired CSF/plasma samples. This is not surprising because as previously reported, glycosylation at this position is highly conserved rendering the virus resistant to broadly neutralizing antibodies by blocking epitopes that can be recognized by these antibodies [43,44]. All glycans at position 160 in the HIV-1 *env* V2 loop (N160) were conserved in all sequences except sequence 1452 from CSF. The removal of this glycan may disrupt the PG16 conformational epitope in the variable loop leading to a decreased neutralization activity of the PG16 antibody [45]. 

Analysis of the HIV-1 V3 loop is important in identifying mutations that may contribute to resistance to entry inhibitors. Mutations A316T and I323V have been reported to contribute to Maraviroc resistance [30]. The selection of 316T has been favoured in HIV-1C strains throughout the years; however, 316A has also been seen as an alternative advantage in some cases [46,47]. We found 61.5% and 60.0% of sequences from plasma and CSF, respectively, harbouring A316T mutation. A high prevalence of this mutation substantiates the finding that A316T is a natural polymorphism found in HIV-1C [48]. We could not establish whether or not this mutation contributes to resistance to entry inhibitors. The prevalence of I323V mutations has been reported to range from 3.8% to 7.1% [48,49]. In our study, we found a prevalence of 8.0% in plasma and 7.7% in the CSF sequences. These mutations were concordant between the CSF and plasma suggesting a lack of variation of HIV-1 *env* V3 loop in the CSF and plasma.

HIV-1 *gag* CTL escape mutations can compartmentalize as a result of positive selection from the host CTL response [20,21]. However, our study showed very little discordance in the HIV-1 *gag* CTL escape mutations between the CSF and plasma suggesting similar CTL selective pressure. Mutation T242N in the TW10 epitope that is commonly known for reducing viral replication capacity was observed in only two sequences. However, compensatory mutations H219Q, I223V and/or M228L, which are associated with T242N mutation were observed in most of our sequences. The occurrence of these compensatory mutations in the absence of T242N may indicate the reversion of T242N to wildtype following transmission to an HLA-mismatched host [50,51]. 

Our study was limited by a relatively small number of participants. A lack of a control (HIV positive individuals without CM) has also been a limitation of this study because we could not confidently conclude on the effects of CM on compartmentalization. Using Sanger-based population sequencing limited our ability to assess minority variants in the two compartments that might also contribute to viral compartmentalization. Therefore, we recommend the use of robust methods such as single genome amplification or next generation sequencing to look in-depth at these genetic variations.

## 5. Conclusions

Our results suggest that there is little variation in HIV-1C *env* derived from CSF and plasma, furthermore, the CCR5-tropic strains predominate in these two compartments even in participants with advanced HIV disease and HIV-associated CM. However, a relatively high proportion of strains harbouring Maraviroc resistance-associated mutations were observed in both compartments leaving a question of whether they will be susceptible to this drug or not. Our study, which was one of the few studies that investigated discordance in the HIV-1 *gag* CTL escape mutations between the CSF and plasma showed little variation in these mutations between the two compartments. Although our results were not significant, they show the importance of carrying out studies that may help to understand the implications of these CTL escape mutations on potential novel treatments, therapeutic vaccines, and HIV cure strategies.

## Figures and Tables

**Table 1:** Baseline characteristics and demographics of the 41 study participants.

Patient Characteristics, N = 41	
Median age, years (IQR)	38 (31–44)
Gender	
Male (%)	59.5
Female (%)	40.5
Median CD4 count, cells/μL (IQR)	28 (10–44)
On ART or previously exposed, *n* (%)	
Yes	21.4
No	78.5
Median Viral load, log_10_ copies/mL	
Plasma	5.1 (4.8–5.7)
CSF	4.6 (3.7–4.9)
CSF WBC, cells/μL (IQR)	10 (3.0–65.8)
Median Fungal burden, log_10_ CFU/mL (IQR)	5.0 (4.1–5.7)

CSF, cerebrospinal fluid; IQR, interquartile ranges; 25th percentile and 75th percentile; WBC, white blood cell count.

**Table 2:** HIV-1 gag cytotoxic T-lymphocyte (CTL) escape mutations in the CSF and plasma of individuals with HIV-associated CM.

Sample ID	Sex	Age	CD4 Count	Fungal Burden	Viral Load	Epitope and Epitope Sequence									Compensatory Mutations
						TL9	GL9	RM9	KK10	NY10	ISW9	KF11	TW10	QW9	
1057CSF	M	27	24	5	4.9	None	-	H28P	None	None	I147L	None	None	None	H219Q, I223V, M228I
1057PLASMA					5.8	None	None	-	None	None	I147L	None	None	None	H219Q, I223V, M228I
1925CSF	F	38	37	5	4.6	Q182H	None	None	None	D260E	None	None	None	None	H219Q, I223V
1925PLASMA					4.9	Q182H	None	None	None	D260E	None	None	None	None	H219Q, I223V
1310CSF	F	23	22	6.2	4.6	**Q182A**	-	-	-	-	I147L	None	None	-	None
1310PLASMA					5.8	**Q182A/T**	None	H28R	L268M	None	I147L	None	None	None	None
1222CSF	F	46	3	5.8	2.8	None	G357S	**H28R**	None	None	None	None	None	A309S, T310S	I223V
1222PLASMA					3.9	None	G357S	**H28R, M30R**	None	None	None	None	None	A309S, T310S	I223V
1326CSF	M	34	10	6.1	4.0	None	None	H28Q	None	None	None	None	None	None	H219Q, I223V
1326PLASMA					5.1	None	None	H28Q	None	None	None	None	None	None	H219Q, I223V
1313CSF	F	30	66	4.6	4.8	None	G357S	None	None	D260E	I147L	None	None	**None**	I223V
1313PLASMA					5.7	None	G357S	None	None	D260E	I147L	None	None	**T310S**	I223V
1134CSF	M	37	222	3.6	2.8	None	None	H28R	None	None	None	None	None	None	H219Q, I223V, M228L
1134PLASMA					4.1	None	None	H28R	None	None	None	None	None	None	H219Q, I223V, M228L
1473CSF	M	39	26	5.1	5.6	None	G357S	None	None	None	I147L	None	None	None	S165N, I223V, M228I
1473PLASMA					5.1	None	G357S	None	None	None	I147L	None	None	None	S165N, I223V, M228I
1121CSF	F	30	8	4.7	5.3	Q182S	G357S	H28Q	None	D260E	None	None	None	None	I223A
1121PLASMA					6.2	Q182S	G357S	H28Q	None	D260E	None	None	None	None	I223A
1500PLASMA	F	42	41	5.9	5.6	None	G357S	H28R	None	None	I147L	None	None	T310S	I223V
1972PLASMA	F	36	N/A	4.1	5.7	Q182S, T186S	None	H28Q	None	None	I147L	None	None	T310S	I223V, M228I
1884CSF	M	48	166	7.0	4.9	Q182A	G357S	-	None	None	I147L	None	None	None	I223V
1452PLASMA	F	39	56	3.6	NA	None	None	None	None	D260E	A146N, I147L	A163G	None	None	S165N, I223V
1801PLASMA	M	37	26	5.7	4.9	None	None	-	None	D260E	I147H	None	None	None	M228I
1164CSF	M	39	53	4.9	2.6	N/A	G357S	-	L268M	None	-	-	T242N	T310S	H219Q, I223A
1730PLASMA	F	35	28	5.9	5.3	None	G357S	H28R	None	None	A146S	None	None	None	I223V, M228L
1362PLASMA	F	30	21	3.7	N/A	None	None	H28Q	None	D260E	I147L	None	None	None	None
1930CSF	M	27	40	5.2	4.9	None	None	H28Q	None	None	A146T, I147L	None	None	None	H219Q, I223V
1862CSF	M	36	37	4.7	5.4	None	G357S	None	None	D260E	I147L	None	None	None	H219Q, I223V, M228I
1527PLASMA	M	44	30	5.3	NA	None	None	H28Q, M30R	None	None	None	None	None	A309S, T310S	H219Q, I223V
1255CSF	F	41	5	5.6	4.6	None	G357S	M30R	None	D260E	I147L	None	None	None	I223V
1724PLASMA	M	38	28	2.7	4.7	None	G357S	None	None	None	None	None	None	None	I223V
1496PLASMA	F	25	8	6.7	4.8	None	G357S	None	None	None	I147L	None	None	None	None
1355PLASMA	M	22	31	2.9	5.0	Q182S	None	M30R	None	None	A146S	None	None	None	I223V
1748PLASMA	M	46	164	0	NA	None	G357S	H28R	None	None	None	None	None	A309S	I223V
1374CSF	F	49	1	4.9	5.0	None	G357S	-	None	None	None	None	T242N	None	H219Q
1352PLASMA	M	42	120	5.6	5.8	None	None	H28Q	None	None	None	None	None	None	I223V

CSF, Cerebrospinal Fluid; F, Female; M, Male; N/A, not available; age (years); CD4 count (cells/μL); fungal burden (log_10_ CFU/mL); viral load (log_10_ copies/mL); dashes mean the region of interest was not covered during amplification and hence could not report any mutations. The results in red signify the mutations between the two compartments.

## Supplementary Materials

The following are available online at https://www.mdpi.com/1999-4915/12/12/1404/s1, Figure S1: Potential N-linked glycosylation sites (PNGS) in HIV-1 env V2 loop in CSF and plasma paired samples. PNGS are highlighted in red. Any sequence that had X as a result of mixed bases at any position analyzed was not considered to have lost the glycan site. Figure S2: Potential N-linked glycosylation sites (PNGSs) in the HIV-1 env V4 loop in CSF and plasma paired samples. PNGSs are highlighted in red. Any sequence that had X as a result of mixed bases at any position analyzed was not considered to have lost the glycan site.
