# Supplementary figures and images for: HIV-1C env and gag Variation in the Cerebrospinal Fluid and Plasma of Patients with HIV-Associated Cryptococcal Meningitis in Botswana
Source: Viruses. 2020 Dec 7;12(12):1404. doi: 10.3390/v12121404 (PMC7762280; doi:10.3390/v12121404)

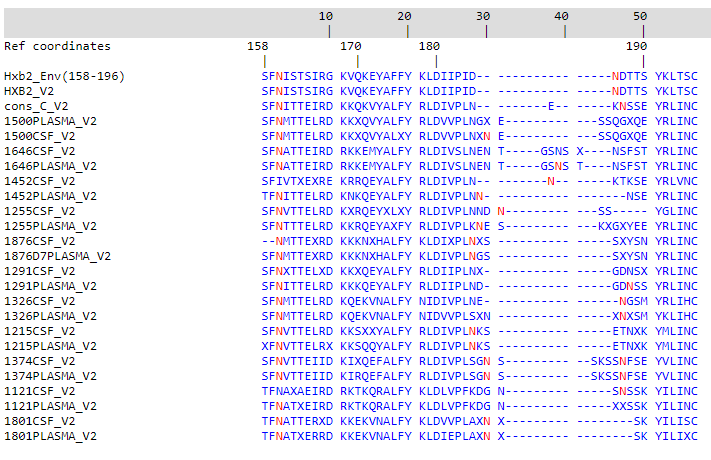

Supplement: Supplementary file 1 [file viruses-12-01404-s001.zip › Supplementary Figure S1.png]

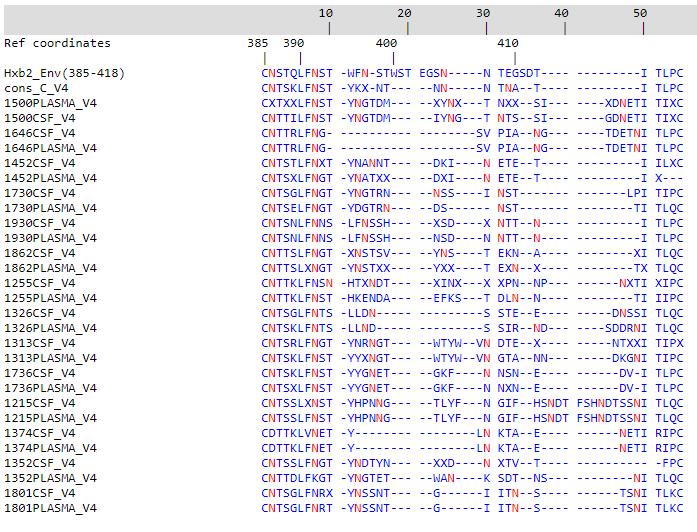

Supplement: Supplementary file 1 [file viruses-12-01404-s001.zip › Supplementary Figure S2.png]
